# MSFF-Net: Multi-Stream Feature Fusion Network for surface electromyography gesture recognition

**DOI:** 10.1371/journal.pone.0276436

**Published:** 2022-11-07

**Authors:** Xiangdong Peng, Xiao Zhou, Huaqiang Zhu, Zejun Ke, Congcheng Pan

**Affiliations:** School of Software and Internet of Things Engineering, Jiangxi University of Finance and Economics, Nanchang, Jiangxi, China; PDPM IIITDM: PDPM Indian Institute of Information Technology Design and Manufacturing Jabalpur, INDIA

## Abstract

In the field of surface electromyography (sEMG) gesture recognition, how to improve recognition accuracy has been a research hotspot. The rapid development of deep learning provides a new solution to this problem. At present, the main applications of deep learning for sEMG gesture feature extraction are based on convolutional neural network (CNN) structures to capture spatial morphological information of the multichannel sEMG or based on long short-term memory network (LSTM) to extract time-dependent information of the single-channel sEMG. However, there are few methods to comprehensively consider the distribution area of the sEMG signal acquisition electrode sensor and the arrangement of the sEMG signal morphological features and electrode spatial features. In this paper, a novel multi-stream feature fusion network (MSFF-Net) model is proposed for sEMG gesture recognition. The model adopts a divide-and-conquer strategy to learn the relationship between different muscle regions and specific gestures. Firstly, a multi-stream convolutional neural network (Multi-stream CNN) and a convolutional block attention module integrated with a resblock (ResCBAM) are used to extract multi-dimensional spatial features from signal morphology, electrode space, and feature map space. Then the learned multi-view depth features are fused by a view aggregation network consisting of an early fusion network and a late fusion network. The results of all subjects and gesture movement validation experiments in the sEMG signal acquired from 12 sensors provided by NinaPro’s DB2 and DB4 sub-databases show that the proposed model in this paper has better performance in terms of gesture recognition accuracy compared with the existing models.

## 1. Introduction

Surface electromyography (sEMG) is a signal graph that uses electrodes to measure muscle electrical activity from the surface of the skin, the recorded signals provide relevant information about human activities. sEMG has important practical value in clinical medicine, ergonomics, and rehabilitation medicine. Human-computer interaction methods based on sEMG are not only widely used in prosthetics [1], sign language recognition systems [2], intelligent driving [3], virtual reality [4], etc. field. The main forms are wearable devices in the consumer field, and the product form is computer games or drone control by wristbands; auxiliary robotic arms in the industrial field; muscle kinematics analysis equipment, prosthetic hands, and sports exoskeletons in the medical and health field. In addition, sEMG also has considerable potential in assessing fatigue during exercise training [5] as well as in muscle type injury detection [6] and stroke rehabilitation [7].

According to the difference in electrode equipment, sEMG can be divided into sparse sEMG and high-density sEMG [8]. Sparse sEMG forms an image according to the signal amplitude in a certain time window, and the two-dimensional electrode array equipment used by high-density sEMG can directly construct an image according to the amplitude of the instantaneous signal [9]. In the review and research of sparse sEMG pattern recognition algorithm [10, 11], the whole process can be divided into three stages: (1) preprocessing. Remove the noise in the original signal and convert the long-time signal into an adaptive format. (2) Feature extraction. Extract high-level semantic features of time, frequency, time-frequency domain, or deep learning network for intention recognition. (3) Classification or regression. Label or number of forecast tasks.

The existing sparse sEMG pattern recognition methods can be roughly divided into two categories: (1) methods based on feature engineering (2) methods based on feature learning [12].

Feature engineering methods improve information quality and density by designing features such as feature extraction time and frequency and then selecting appropriate classifiers to complete the gesture recognition task. Such as linear discriminant analysis (LDA) [13], principal component analysis (PCA) [14], support vector machine (SVM) [15], random forest (RF) [16], and the k-nearest neighbor algorithm (k-NN) [17, 18]. These methods achieve good results on the recognition task for a small number of gestures, but the recognition rate decreases significantly as the number of recognized gestures increases. Moreover, finding the best feature collection is a very time-consuming task, which requires professional knowledge and experience, and its generalization performance is poor. Constructing a model that can automatically extract sEMG features for classification is the key to improving the recognition rate of multi-gesture classification.

In the feature learning method, features are automatically generated by the machine learning algorithm, therefore, the research focus has shifted from manual feature engineering to automatic feature learning. In the research on improving the accuracy and real-time performance of gesture prediction, Wei et al. [19] combined the traditional feature set with the convolutional neural network (CNN) model based on deep learning, and used multi-view learning to sEMG signal, and achieved good results on the NinaPro database. However, the evaluation and combination experiments of 11 feature sets are more complex, which does not better reflect the advantages of automatic feature extraction by deep learning. Tsinganos et al. [20] made use of the time series characteristics of sEMG and added a time convolution network and attention mechanism and achieved a good recognition effect. However, the model needs the whole sEMG gesture sequence, and the activity duration in real life is uncertain and lacks practicability. Rahimian et al [21] used expanded convolution to classify upper limb gestures, but the one-dimensional convolution network can only extract features along one direction of time or electrodes and did not make full use of the shape of different time signals in sEMG and the information of electrode space. Wei et al. [22] divided the data of different time frames and adjacent time frames into branches to extract the feature changes of different temporal actions but did not further test the effect of joint actions between electrode sensors on the recognition performance.

In summary, there is room to improve the recognition accuracy of deep learning-based methods on sparse EMG signals. Inspired by the studies above, we propose an sEMG gesture recognition model based on a multi-stream feature fusion network (MSFF-Net) on the DB2 and DB4 sub-datasets of the publicly available dataset NinaPro, which focuses on improving the accuracy of gesture recognition. In this model, we enrich the idea of the multi-stream convolution network and focus on the information of different muscle regions with three branches. The model uses a multi-stream convolutional neural network (Multi-stream CNN) and a convolutional block attention module integrated with a resblock (ResCBAM) to alternately extract the morphological features of signals in different periods and the spatial features of the different number of electrode acquisition channels. Then, the early signal features and the deep features after the late multi-stream convolution network are fused in proportion and sent fusion features to the classifier to output the classification results. Experiments show that this method is superior to other existing methods in experimental data processing and recognition accuracy.

The major contributions of this paper are summarized as follows:

1. We propose a method to analyze multi-channel sEMG signals separately according to muscle regions, which reduces the influence of different muscle regions in feature extraction and strengthens the connection of signals in the same region.
2. A novel MSFF-Net model for sEMG gesture recognition is proposed. Combined with the characteristics of the sEMG signal, the model extracts and fuses the features of the sEMG signal from the aspects of signal morphological features, electrode spatial features, and early-late stage feature fusion.
3. We developed an experiment for the proposed model using the sEMG signal obtained by 12 sensors provided by NinaPro’s DB2 and DB4 sub-databases. Compared with similar methods, it has better recognition accuracy.

The rest of this paper is organized as follows. In Section II, we introduce the sEMG signal preprocessing and describe the proposed MSFF-Net in detail. Section III presents the experiment process and the results of the proposed method. Section IV discusses BN layer order effect, early and late network weight effects, ablation studies, and a comparison of similar literature. Section V finally concludes our work.

## 2. Methods

In this part, we will first introduce the multi-stream feature fusion network overall framework for sEMG gesture classification, then introduce the steps and methods of sEMG signal preprocessing, and finally introduce the composition of the multi-stream feature fusion network in detail.

### 2.1 The overall framework

**Fig 1:**
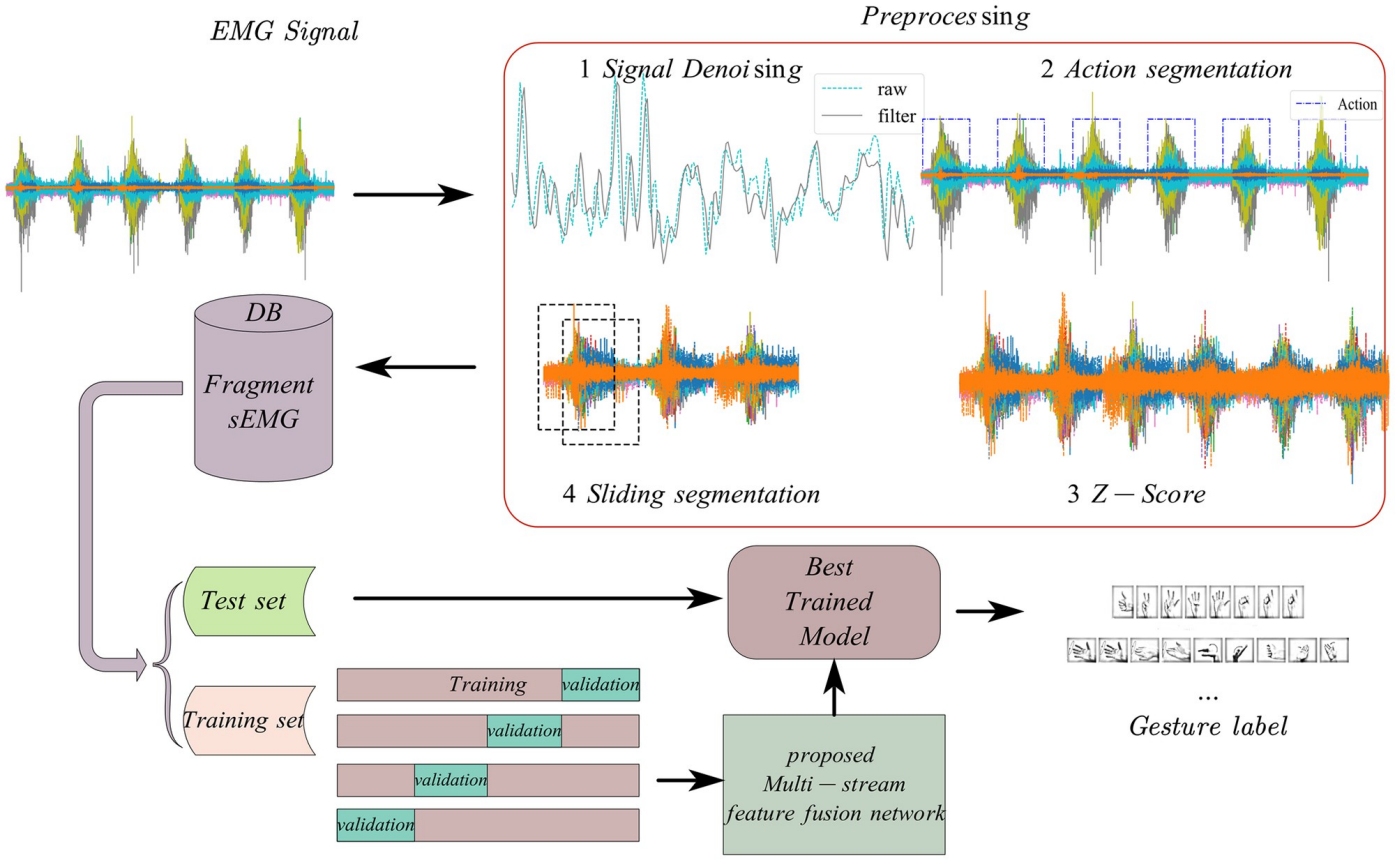
The sEMG gesture recognition overall framework based on MSFF-Net.

The overall frame diagram (Fig 1) shows the complete flow of our proposed multi-stream feature fusion network-based sEMG signal gesture classification. The data used in this article come from Ninapro DB2 and DB4 databases. More than half an hour of raw data per subject in the database needs to be preprocessed to fit the network input, including denoising, action segmentation, data normalization, and fragment EMG generation. The preprocessed data is divided into datasets and trained in the network by cross-validation. At the end of the training, the best model on the validation set is kept for testing on the test set.

### 2.2 Preprocessing

The sEMG is a nonlinear non-stationary time-series signal that can reflect information related to muscle and body behavior, generated by weak action potentials generated by muscle fibers on the skin surface when the skeletal muscle contracts. Like other physiological electrical signal measurements, they are easily corrupted by noise. Three types of noise appear: power-frequency interference, white Gaussian noise, and baseline wander, making sEMG signals difficult to analyze and having a low signal-to-noise ratio. To better analyze the sEMG signal, preprocessing is required. The preprocessing process mainly includes denoising, action segmentation, normalization, and data segmentation.

#### 2.2.1 Denoising

The amplitude of the sEMG signal collected by the electrode sensor is usually between 15 and 100 *μ*V, and the energy of the useful signal is mainly distributed between 10 Hz and 500 Hz [23, 24]. At present, the collection equipment on the market has a certain filtering effect. In this paper, the fourth-order Butterworth filter is used for band-pass filtering, the pass-band boundary is 10~500 Hz, and the sEMG signal is simply denoised.

#### 2.2.2 Action segmentation

The filtered EMG signal retains the main energy part of the signal. The goal of sEMG signal pattern recognition is to identify specific actions that are in demand, and some studies have proposed active segment detection algorithms for signals [25]. For databases without action start times, an algorithm can be used for active segment detection. For the NiproDB2 database with movement activity segments labeled, we segmented each movement of each subject with the resting state as a separation, and we can obtain 49 action categories × 6 repetitions = 294 complete gestures.

**Fig 2:**
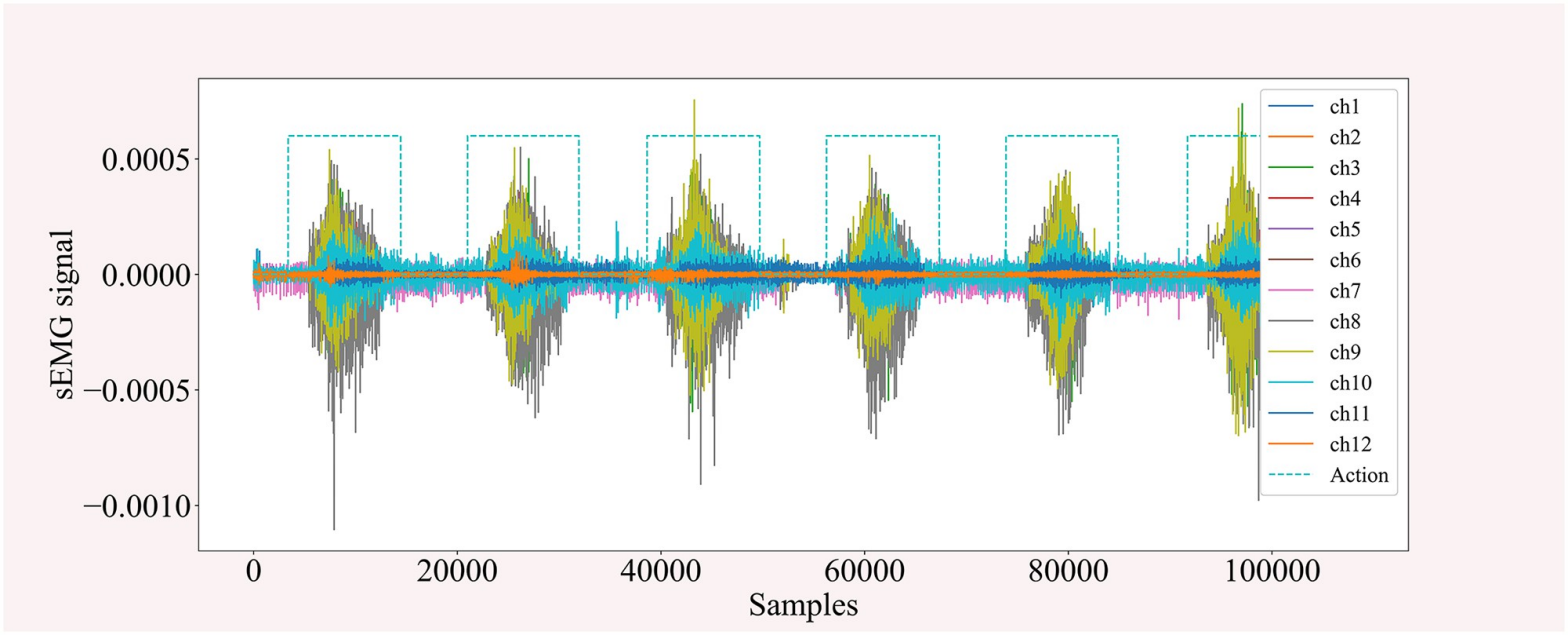
A certain type of action EMG of subject 1.

Fig 2 shows six repetitions of a certain type of gesture recorded for subject 1 acquired by 12 electrodes. During the action segmentation, the duration of each action is different due to the high frequency of the acquisition device and the difference in the labeling method as shown in Fig 2. To fit the input format of the deep learning network, it is also necessary to further partition the actions into uniform fragment sEMG.

#### 2.2.3 Z-Score normalization

The data value of the sEMG signal collected after filtering and motion segmentation is extremely small, and the difference between the data is generally 100 times, which directly affects the experimental results. Normalization algorithms such as Min-Max normalization, Z-score normalization, or conversion to a fixed range are usually used. Our experiments achieve good results on Z-Score normalization. Its mathematical formula is as follows:

Convert all segmented sEMG motion data *x*_1_, *x*_2_, *x*_3_, …, *x*_*n*_ independently by electrode channel:

$$y_{i}=\frac{x_{i}-\mu }{\sigma }$$ (1)

*μ* is the mean of the population data for a single electrode channel, *σ* is the standard deviation of the overall data for a single electrode channel. Z-Score normalization was performed on the 12-channel sEMG data in turn. The data of 400 sampling points with a duration of 200ms were selected for comparison.

**Fig 3:**
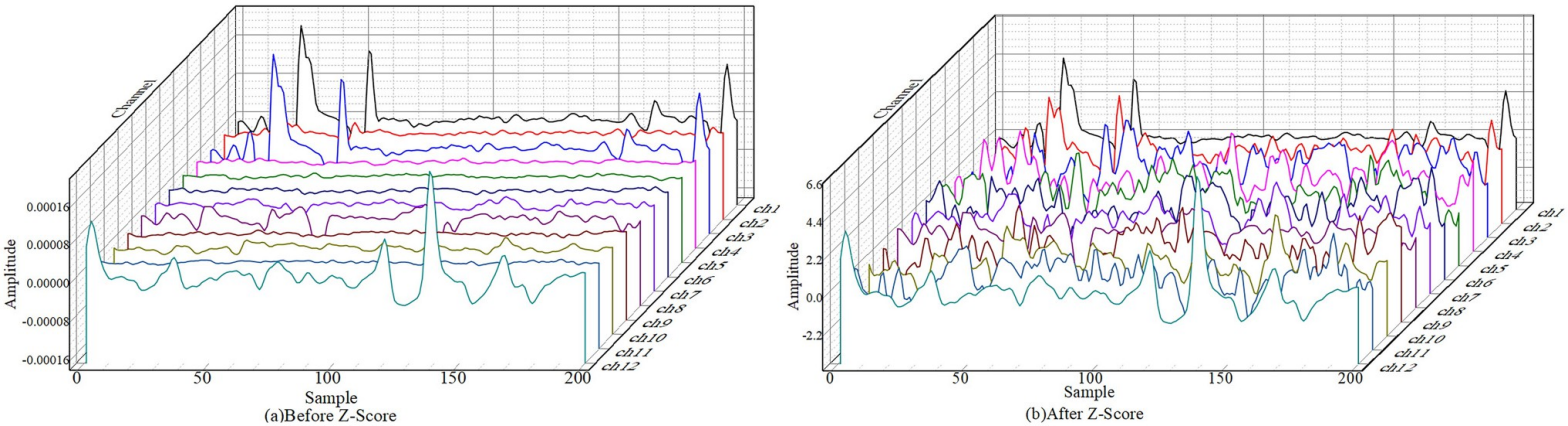
Z-score standardized comparison chart.

As shown in Fig 3, the x-axis represents the sampling of different time frames, the y-axis represents different electrode channels, and the z-axis represents the amplitude of the signal. Z-Score normalization normalizes the value of the real signal from 10^−4^ to around 1, this process preserves the same electrode channel signal distribution, reduces the influence of outliers, and concentrates the data into more easily distinguishable intervals.

#### 2.2.4 Fragment EMG generation

**Fig 4:**
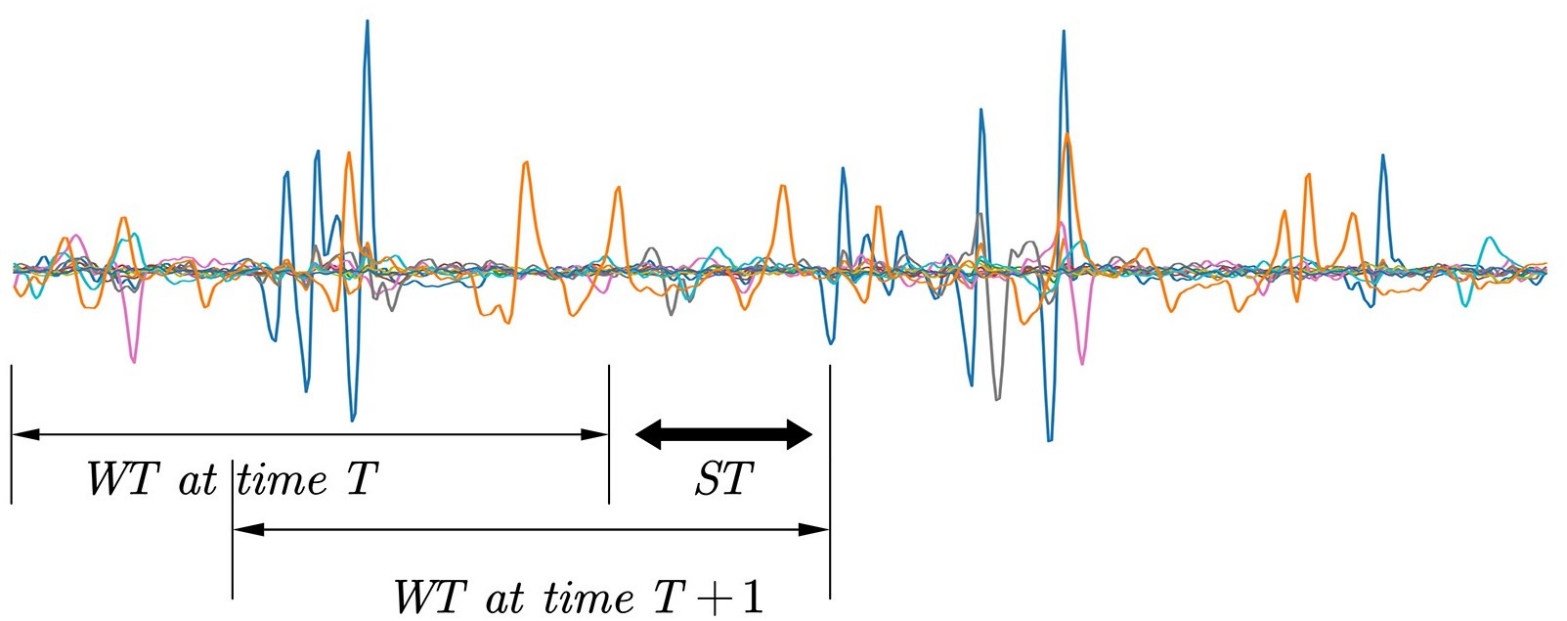
Schematic diagram of sliding segmentation.

After the sEMG signal is normalized, we decompose it into small window segments using a sliding window strategy and an overlapping window scheme to fully utilize the computational power of the system. To compare our proposed method with previous work, we follow the segmentation strategy in the former study [19, 26]. For NinaProDB2, the sliding window length (it is marked as ST in Fig 4) is fixed at 200ms, and the step window length (it is marked as WT in Fig 4) is set at 50ms. The sliding segmentation process is separately segmented according to the obtained 294 actions to ensure the independence of each action.

The fragment EMG obtained by sliding segmentation is denoted as $h\epsilon R^{T\times E}$, T is the number of time frames, and E is the number of acquisition electrodes. we take $h\epsilon R^{400\times 12}$, that is, 12 electrodes 400 times of sampling data.

### 2.3 The multi-stream feature fusion network

#### 2.3.1 Network structure

**Fig 5:**
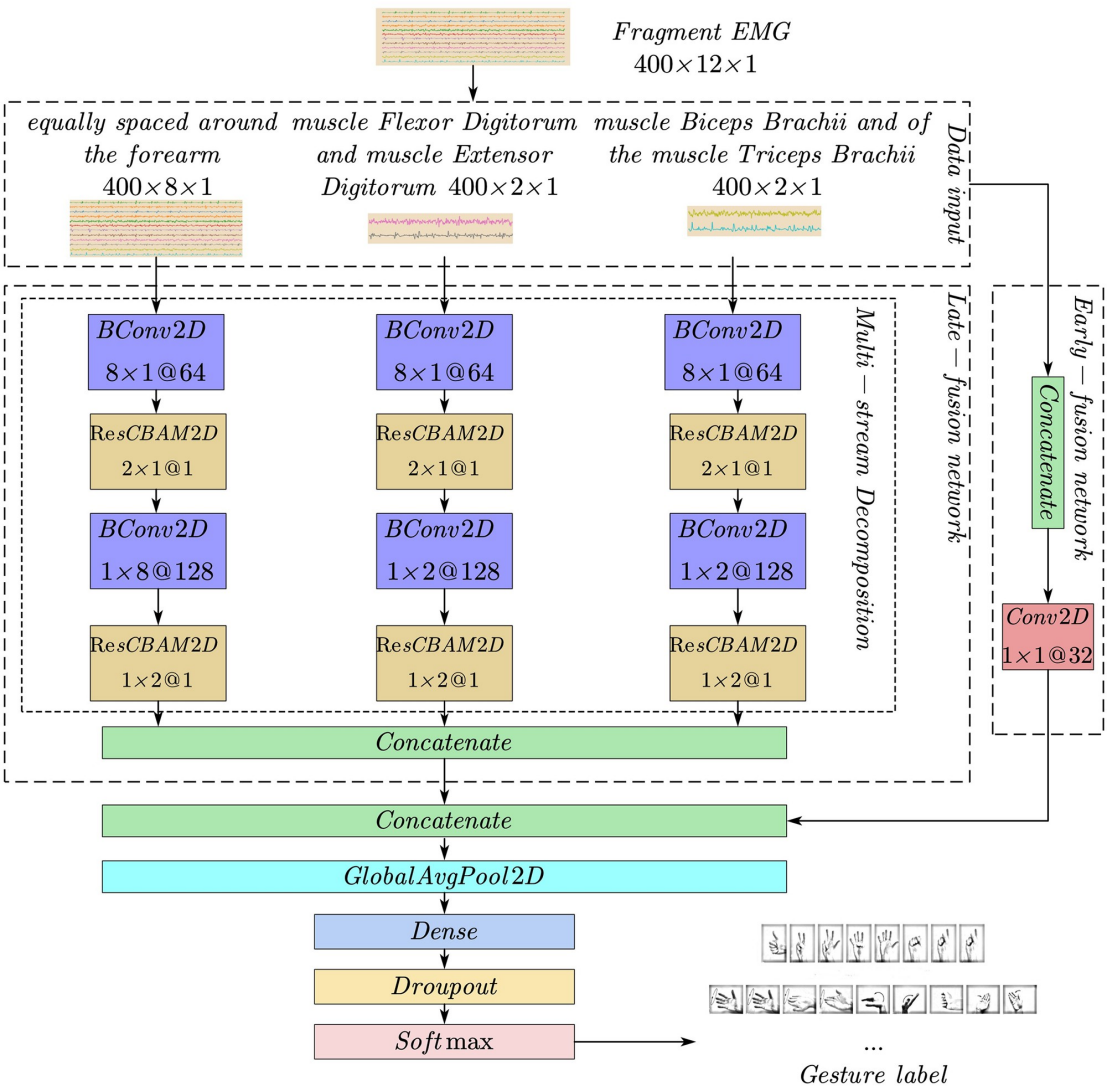
The proposed MSFF-Net model.

The MSFF-Net model proposed in this paper is used for gesture recognition of EMG signals. The model structure is shown in Fig 5. The fragment EMG can achieve end-to-end action recognition on the input signal after supervised training of the network.

The overall network can be divided into three stages, data input stage, multi-stream convolution stage, and global feature aggregation output stage.

The data entry stage for each fragment EMG is represented as $h\epsilon R^{T\times E}$, In the experiment, we found that the two-dimensional convolution method of treating the fragment EMG as a single-channel grayscale image is more effective than the one-dimensional convolution of time series. Two-dimensional convolution can be convolved in two directions. Taking Fragment EMG as an example, the convolution along the T direction can obtain the morphological characteristics of the signal of a single electrode channel, and the convolution along the E direction can obtain different electrode channels and spatial characteristics. Therefore, the dimension enhancement operation is performed on the Fragment EMG, and the new Fragment EMG after the dimension increase is obtained as $h\epsilon R^{T\times E\times C}$, *T* (Time) can be regarded as the length of the image, *E* (Electrode) can be regarded as the width of the image, *C* (Channel) is the number of feature channels, and *C* = 1 means that the image is a single feature channel. The Fragment EMG after the dimension increase avoids the loss of information caused by the compression of the feature information matrix between different electrodes into vectors during the convolution process.

The new combination of images referenced the location of the electrode acquisition: eight electrodes were equally spaced around the forearm, two electrodes were placed on the flexor digitorum and extensor digitorum superficialis, and two electrodes were placed on the biceps and triceps [27]. We divided the input data into three inputs by electrode distribution $h_{1}\epsilon R^{400\times 8\times 1},\;h_{2}\epsilon R^{400\times 2\times 1},h_{3}\epsilon R^{400\times 2\times 1}$, Each input stream focuses on the features of different muscles. The multi-stream convolution stage has three convolutional network branches, corresponding to the three data input streams. That is, different input streams use separate CNN networks for feature extraction.

The role of the multi-stream convolution stage is to extract high-level semantic features. Each branch contains batch normalized convolution modules and residual convolution attention mechanism modules. The batch normalization convolution module is mainly composed of a convolution layer, a ReLU activation layer, and a batch normalization layer. The residual convolutional attention module consists of feature channel attention, spatial attention, and residual modules.

The global feature aggregation output stage consists of two sub-networks: an early fusion network and a late fusion network. As shown in Fig 5, the early fusion network re-integrates the three input data streams into the Fragment EMG after the dimension increase, The data without segmentation and multi-layer convolution retains the early original features and then increases the number of feature maps through 32 1×1 convolution kernels to obtain the output $H_{early}{\in R}^{T\times E\times C}$ (*T* = 400, *E* = 12, *C* = 32). The late fusion network fuses the outputs of the last layer of the multi-stream convolution, extracts the high-level semantic features of the data, and obtains the output $H_{late}{\in R}^{T\times E\times C}$ (*T = 400*, *E = 12*, *C = 128*). Finally, the output of the early fusion network and the late fusion network is aggregated by the feature channel Concatenation to obtain the global feature fusion network layer and the output $H_{final}{\in R}^{T\times E\times C}$ (*T = 400*, *E = 12*, *C = 160*).

The second layer is the global mean pooling layer, which adds and averages the pixel values of each feature channel, and outputs a neuron for each channel to represent the corresponding feature map. *H*_*final*_ gets the output $H_{GAP}\in R^{T\times C}$ (*T* = 400, *C* = 160) after passing through the global mean pooling layer.

The third layer is the fully connected layer, which re-assembles the local features into a complete graph through the weight matrix, and then adds Dropout to prevent overfitting. The last layer uses the fully connected layer of the Softmax activation function to obtain the final classification result $H_{out}\in R^{n}$ (*n* = number of gesture categories). This layer obtains a label vector with a length equal to the number of gesture categories through the fully connected layer, and then the Softmax function predicts the category probability distribution of the label vector. Finally, the result with the highest probability of obtaining a vote is used as the predicted category.

#### 2.3.2 Batch-normalization convolutional module

In the multi-stream convolution stage, we adopt two batch-normalization convolutional modules (BConv). Contains convolutional layers, ReLU activation layers, and batch normalization layers (BN).

The Fragment EMG $h\epsilon R^{T\times E\times C}$ (*C* = 1) composed of multiple electrode channels can be regarded as a grayscale image of a single feature channel. In the batch normalization module, we learn the high-level semantic features hidden by the fragment EMG with 2D convolutional kernels. For the convolutional layers, we use narrow and long convolution kernels that are different from the standard size. Taking the first branch as an example, the size of the convolution kernel is 8×1, which means that each convolution obtains 8 sampling points along the T direction, and obtains the data of one adjacent electrode sampling channel along the E direction, that is, within 4ms of a single collection channel. This process separates different electrode samples, focusing on the morphological characteristics of the signal of a single electrode sample at different periods. The size of the convolution kernel is 1 × 8, which means that each convolution obtains 1 sample along the T direction, and obtains the adjacent 8 electrode sampling channel data along the C direction, that is, the sampling data of the 8 electrodes at the same time. This process separates data at different times and links different electrode sampling channels. Each convolution kernel generates a corresponding feature map.

The convolutional data is activated by ReLU to make the network sparse and reduce the interdependence between parameters, which alleviates the occurrence of overfitting. After the data is processed by the BN layer, it is closer to the origin, so that the activation function in the convolution process of the next layer can obtain a larger gradient, and at the same time, the sparsely distributed data after activation is more closely linked. Data that is closely related is more likely to be fit by machine learning features.

#### 2.3.3 CBAM module with residuals

In recent years, the Attention Model has gradually become an important concept in neural networks. By imitating the idea of human visual attention, applied research has been carried out in different application fields. The convolutional block attention module (CBAM) is an improved model based on CNN and attention mechanism, including two sub-modules of channel attention structure (CAS) and spatial attention structure (SAS). It was proposed and applied to image classification in 2018 [28], and the experimental results confirmed that CBAM outperforms all other methods on three different benchmark datasets. It is proved that the CBAM module is of great significance to improve the performance of the recognition model.

**Fig 6:**
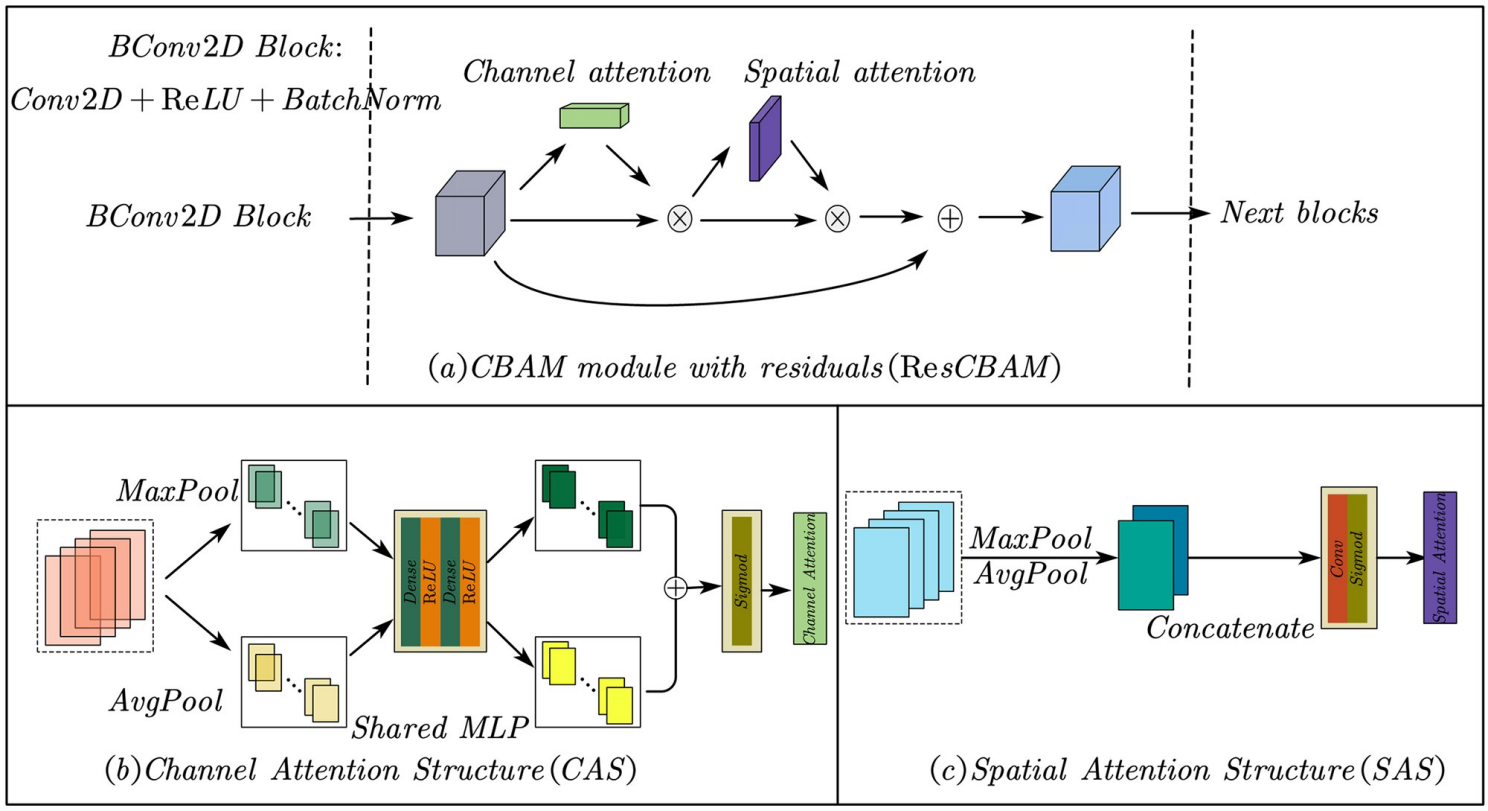
The network structure of the CBAM module with residuals, CSA, and SAS.

The CBAM module with residuals structure is shown in Fig 6(a). From a spatial perspective, channel attention is global, while spatial attention is local. In the experiment, we combined CBAM with ResNet, for the sequential arrangement of the two submodules, the existing experimental results show that the feature channel first is slightly better than the space first [28].

Table 1 shows the detailed network structure of the CBAM module with residuals of the first data input branch.

**Table 1:** The CBAM module with residuals detailed network structure.

Name		Layer	Output Shape	Activation function	Connected to
CBAM module with residuals		Input	(400,8,64)	-	BConv2D
	CAS	Maxpool	(1,1,64)	-	Input
		Avg_pool	(1,1,64)	-	Input
		Dense	(1, 1, 32)	ReLU	Max_pool (Avg_pool)
		Dense_1	(1, 1, 64)	ReLU	Dense (Dense [2])
		Add	(1,1,64)	-	Dense_1 Dense_1 [2]
		Activation	(1,1,64)	Sigmoid	Add
		Multiply	(400,8,64)	-	Input CAS
	SAS	Max_pool_1	(400,8,1)	-	Multiply
		Avg_pool_1	(400,8,1)	-	Multiply
		Concate_1	(400,8,2)	-	Max_pool_1 Avg_pool_1
		Conv2d_1	(400, 8, 1)	-	Concate_1
		Activation	(400, 8, 1)	Sigmoid	Conv2d_1
		Multiply_1	(400, 8, 64)	-	Multiply SAS
		Add	(400,8,64)	-	Multiply_1 Input

[2] indicates the second execution.

() indicates that the network layer input is shared.

Considering the particularity of the Fragment EMG $h\epsilon R^{T\times E\times C}$ compared to the RGB image $h\epsilon R^{H\times W\times C}$, we made some improvements to CBAM. The feature channel attention part is reserved for extracting information between different feature map channels, and the weights of different feature maps are changed through the feature channel attention module, giving more weight coefficients to useful feature maps, and useless feature maps. It is suppressed to a certain extent, and its structure is shown in Fig 6(b).

We follow the approach of Woo et al. [28] and utilize max pooling and average pooling outputs in the feature channel attention sub-module. First, use the average pooling and max pooling operations to aggregate the spatial information of the feature maps to generate two different feature space descriptors $F_{avg}^{C}$ and $F_{max}^{C}$. These two feature space descriptors are sent to a shared network to generate our Channel attention $M_{c}\in R^{1\times 1\times C}$. The shared network consists of a multilayer perceptron (MLP) and a hidden layer. To reduce parameter overhead, the hidden activation size is set to $R^{1\times 1\times C/r}$, where r is the reduction rate. After the shared network is applied to each descriptor, we merge the output feature vectors using element-wise summation. The channel attention is calculated as follows:

$$\begin{array}{ll} M_{c}\left(F\right) & =\sigma (MLP\left(AvgPool\left(F\right))+MLP\left(MaxPool\left(F\right)\right)\right)\\ & =\sigma (W_{1}\left(W_{0}\left(F_{avg}^{C}\right)\right)+W_{1}\left(W_{0}\left(F_{max}^{C}\right)\right) \end{array}$$ (2)

where σ represents the sigmoid function, $W_{0}\in R^{C/r\times C}$ and $W_{1}\in R^{C/r\times C}$. The two inputs share the MLP weights *W*_0_ and *W*_1_.

Spatial attention focuses on the location information of information on feature maps, which is complementary to feature channel attention. To compute spatial attention, we apply average pooling and max pooling operations along the feature channel axis and concatenate the two to generate efficient feature descriptors. Applying pooling operations along the feature channel axis can effectively highlight informative regions [29]. On the concatenated feature descriptor, we use convolution to generate the spatial attention map $M_{s}(F)\in R^{H\times W}$. The spatial attention structure is shown in Fig 6(c). The detailed operations are as follows: First, generate two two-dimensional maps through two merging operations to aggregate the channel information of a feature map: $F_{avg}^{S}\in R^{H\times W\times 1}$ and $F_{max}^{S}\in R^{H\times W\times 1}$. They represent the mean pooling features and max-pooling features in the feature channel, respectively. Then, they are connected and convolved through a standard convolutional layer to generate a 2D spatial attention map, calculated as follows.

$$\begin{array}{ll} M_{s}\left(F\right) & =\sigma \left(f^{a\times a}\left(\left[AvgPool\left(F\right);MaxPool\left(F\right)\right]\right)\right)\\ & =\sigma \left(f^{a\times a}\left(\left[F_{avg}^{s};F_{max}^{s}\right]\right)\right) \end{array}$$ (3)

Where *σ* represents the sigmoid function, *f*^*a*×*a*^ represents the convolution kernel size *a* × *a* is the convolution process.

We changed the size of the *a* × *a* convolution kernel in the spatial attention part based on image processing, using a 2×1 convolution kernel to extract the morphological features of adjacent temporal signals, and a 1×2 convolution kernel is used to extract the spatial features of adjacent electrode acquisition channels. This process corresponds to the Conv2D network layer of the SAS module in Table 2. Additionally, we replace max and mean pooling in this process with global max pooling, global mean pooling, and reshape operations.

**Table 2:** The Ninapro database summary table.

	NinaproDB2	NinaproDB4
**Number of classified gestures**	49	52
**Intact subjects**	40	10
**Electrodes**	12 Delsys	12 Cometa
**Sampling rate**	2000Hz	2000Hz
**Number of trials**	6	6
**Exercise1**	Exercise B	Exercise A
**Exercise2**	Exercise C	Exercise B
**Exercise3**	Exercise D	Exercise C

## 3. Experiments and results

### 3.1 Database

In this study, NinaPro databases DB2 and DB4, which contain tasks related to upper extremity movement, were used for the experiments. Table 2 summarizes the information and descriptions of the two databases.

NinaPro is a publicly accessible database that has previously been used for myoelectric interface implementations to decode human hand movements. The DB2 sub-database collected sparse sEMG data from 40 healthy subjects including 11 females and 29 males using 12 Delsys wireless electrodes on the subject’s forearm surface and filtered through a Hampel filter to eliminate 50 Hz power frequency interference [27]. The DB4 sub-database collected sparse sEMG data from 10 healthy subjects including 6 males and 4 females using 12 Cometa wireless electrodes on the subject’s forearm surface [30].

**Fig 7:**
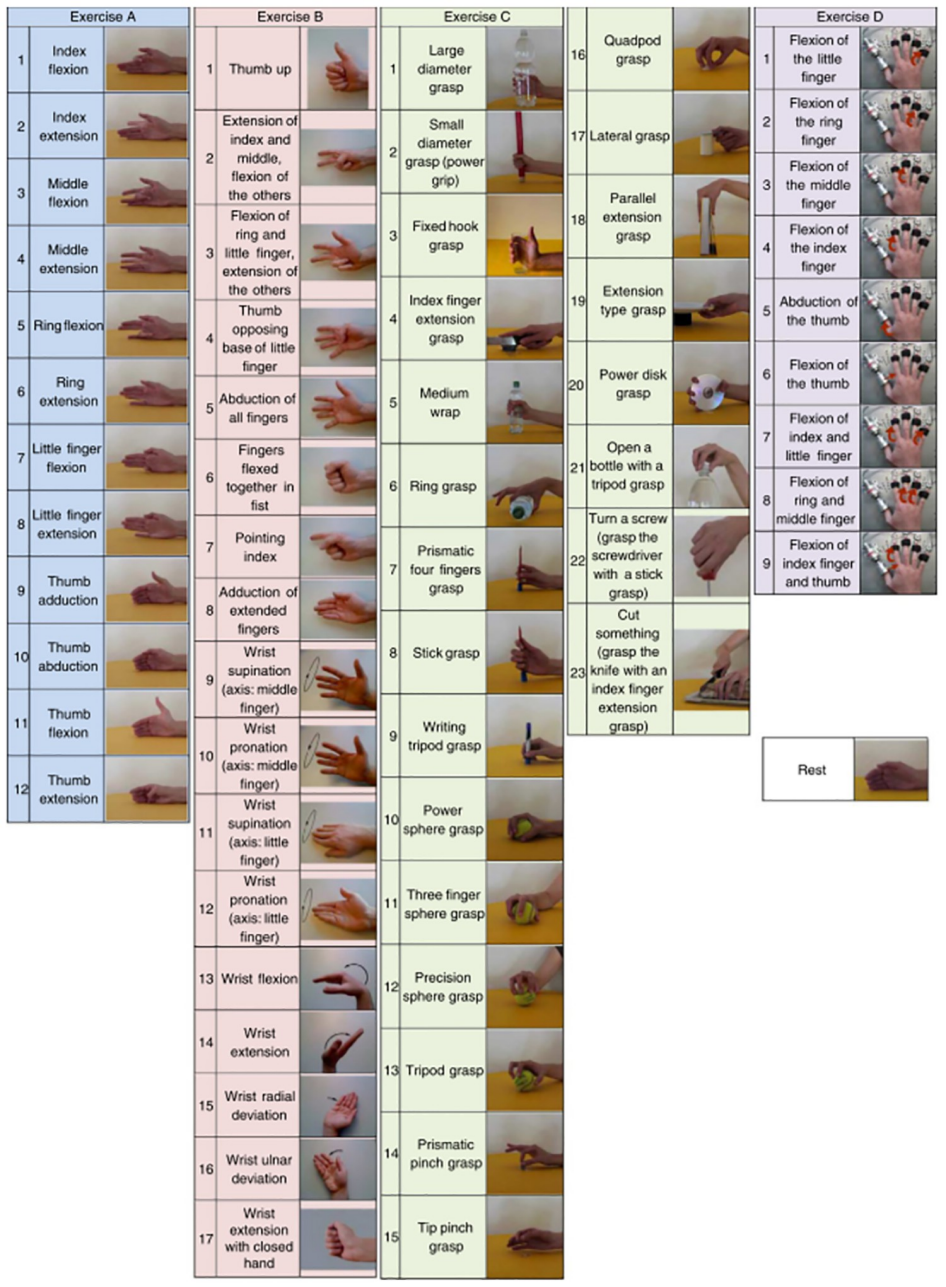
The Ninapro database gesture action chart.

Both databases follow the same experimental acquisition protocol, and the captured gesture actions are divided into 3 exercises, the detailed actions of which are shown in Fig 7. Each movement was repeated 6 times, with each exercise lasting 5 s and alternating with a resting position lasting 3 s. The biggest difference between the two is that the data collected by DB2 is at the microvolt level (*μ*V), and the data collected by DB4 is at the volt level (V) after amplification.

**Fig 8:**
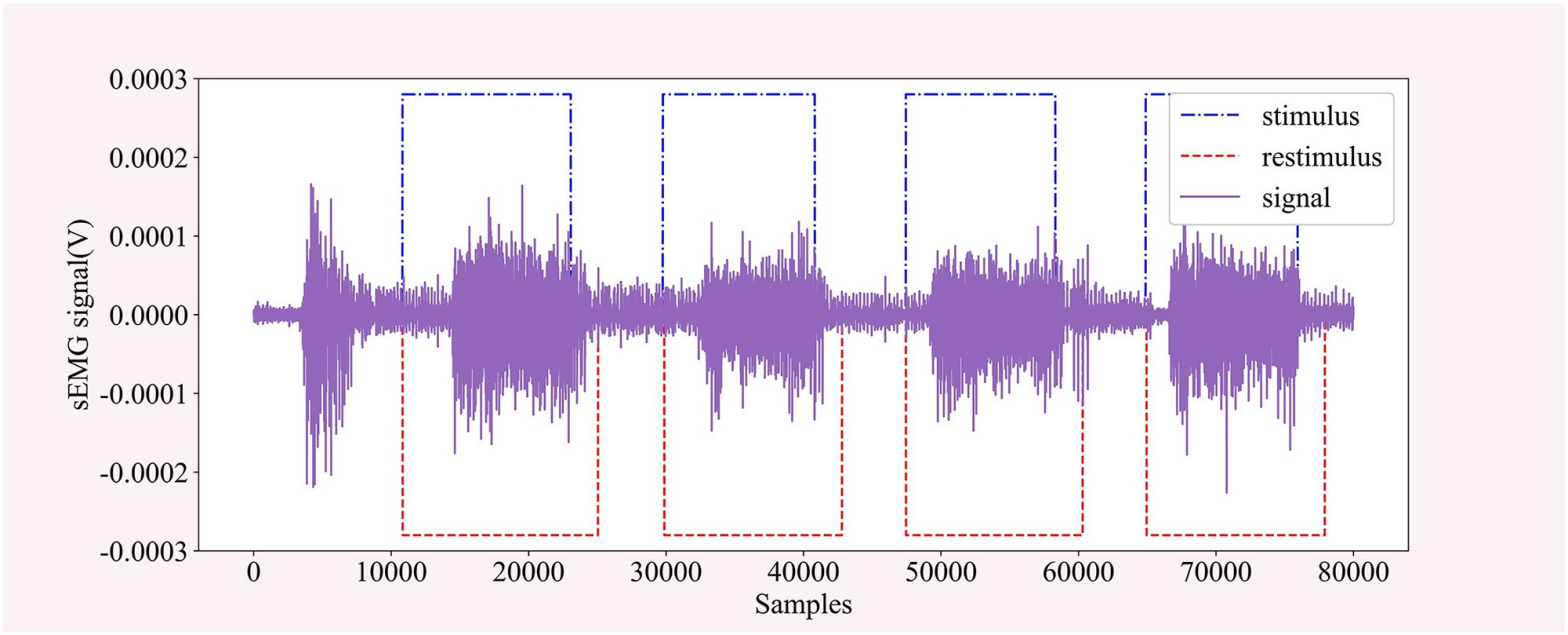
The schematic diagram of stimulus and restimulus label.

It is worth mentioning that the Ninapro database has two sets of movement classification labels and corresponding movement repetition labels; the stimulus records the labels generated for each sample using the stimulus generator; the restimulus records the posterior labels of the movements. The processes associated with movement durations in the posterior labels are refined to represent real movements [31]. It is shown in Fig 8.

The repetition is temporally synchronized with the stimulus recordings, recording the number of repetitions and the duration of each action, and can be used to do segmentation of the active segment of the signal. We experimentally did both kinds of data separately considering the differences in labeling, which is something that has been rarely mentioned by others except Rahimian et al [21].

For dataset partitioning, we follow the dataset partitioning strategy in the former study [19, 26]. After action segmentation, according to the number of repetitions of each action, the 1st, 3rd, 4th, and 6th repetitions are used as the training set, and the 2nd and 5th repetitions are used as the test set.

**Fig 9:**
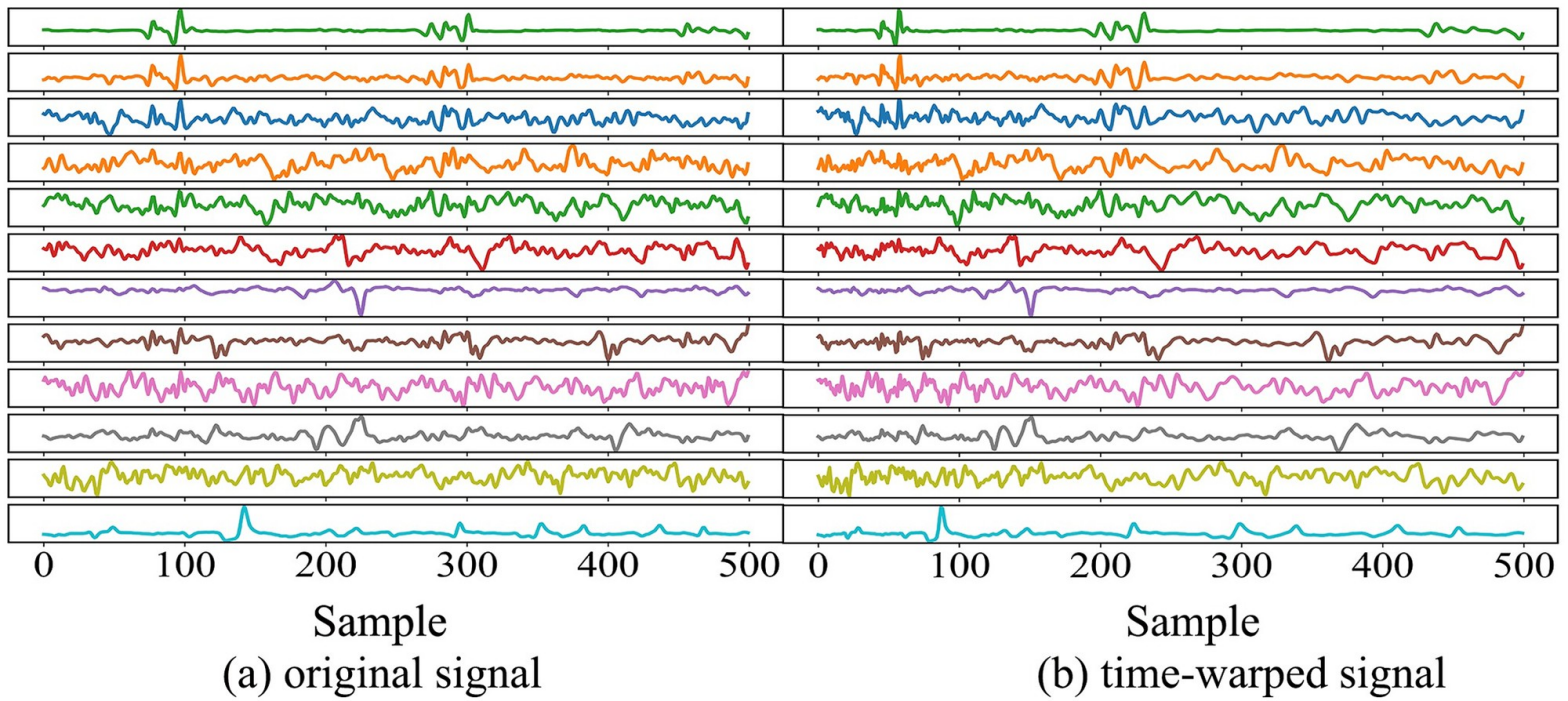
The enhanced comparison of time-warping data.

Due to the small number of complete gestures in each category in the Ninapro database and the sparsity of EMG signals, overfitting is easy occurs. Furthermore, the amplitude and duration of sEMG cannot be fully replicated during motion repetition acquisition, we time-warped the training set data [32] and expanded the training data to twice the original size. The time-warped data enhancement comparison chart is shown in Fig 9.

We take the data of 500 samples as an example. The time-warped data randomly changes the timeline of the original data. The degree of time-warping is controlled by the number of speed changes and the ratio of the maximum/minimum speed. The warped data retains the difference between different channels. real-time and signal amplitude. We take the data of 500 samples as an example. The time-warped data randomly changes the timeline of the original data. The degree of time warping is controlled by the number of speed changes and the ratio of the maximum/minimum speed. The time-warped data preserve the synchronization and amplitude between the different electrodes.

### 3.2 Evaluation metric

We adopted the same intrasubject schemes as those were most commonly used in existing studies on the NinaPro database [9, 33, 34]. In intrasubject evaluation, the deep learning model is trained on a part of the data from one subject and tested on the non-overlapping part of the data from the same subject. We follow this evaluation scheme, specifically, we used repetitions 1,3,4, and 6 for training purposes and repetitions 2 and 5 for testing. The final gesture recognition accuracy is obtained by averaging the accuracy achieved by all subjects.

Classification Accuracy: Classification accuracy is defined as the ratio between the number of correctly classified gesture segments in a trial and the total number of gesture segments tested. The Accuracy (Acc) of the target object is calculated as follows:

$$Acc=\frac{Number\;of\;correct\;classifications}{Total\;number\;of\;test\;samples}$$ (4)

Overall classification accuracy: Overall classification accuracy (Overall Accuracy, OA) is defined as the average of the classification accuracies of all experimental individuals and is calculated as follows.

$$OA=\frac{1}{M}\sum_{n=1}^{M}Acc$$ (5)

where M is the number of subjects.

### 3.3 Experimental setup

The network proposed in this paper is implemented based on Keras with Tensorflow as the backend and is trained using RTX2080ti. The loss function uses the cross-entropy function, and the model is trained using the Adam optimization algorithm. The number of training sessions is set to 50 epochs, and the learning rate is set to 0.001. During the training process, the model with the highest validation set Accuracy will be saved as the final model.

### 3.4 Experimental results

Our proposed method follows the dataset partition of the former study. To compare with more researchers, we use the repeated gesture cross-validation method to conduct experiments on the stimulus data and the restimulus data respectively, and the result is taken as the average of the overall recognition accuracy in multiple experiments. As shown in Table 3. The cross-validation results can test the generalization ability of the network.

**Table 3:** The classification results of DB2 and DB4 stimulus data and restimulus data.

Database	Fold 1	Fold 2	Fold 3	Fold 4	Average
DB2 Stimulus OA (%)	86.83	87.44	87.09	86.72	87.02
DB2 Restimulus OA (%)	85.38	86.00	85.80	85.41	85.65
DB4 stimulus OA (%)	83.23	85.5	85.43	85.31	84.87
DB4 Restimulus OA (%)	83.48	84.71	84.55	84.23	84.24

The results in Table 3 show that our network can achieve better classification results when there are differences in data annotation. Unexpectedly, the recognition accuracy of the stimulus label data is higher than that of the restimulus label data. The reason is that although the restimulus label is more in line with human motion during the data labeling process, the actual labeling time of some gestures is much higher or lower than the standard test time, which makes the data imbalance between gesture actions and affects the result. The same result was confirmed in Rahimian et al. [21].

**Fig 10:**
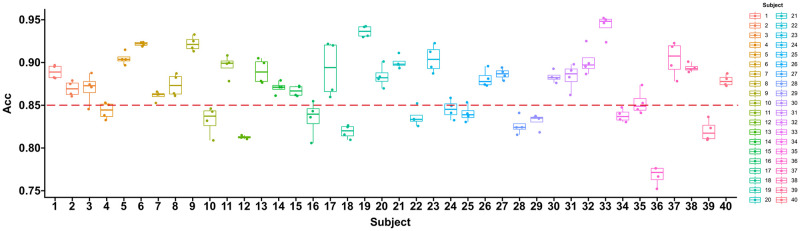
The average gesture recognition accuracy of DB2 subjects.

To compare the differences between different subjects, we show the average recognition accuracy of 49 categories of actions for 40 subjects during four cross-validation processes on the DB2 database. As shown in Fig 10.

The smaller box of the subject cross-validation results indicates that our network can generalize to the recognition of repeated gestures from the same subject. To further analyze the reasons for the discrepancy in recognition accuracy, we combine the attributes already given by the database (Table 4) and take 85% accuracy as the baseline. Marks higher than the baseline were marked as high, otherwise marked as low.

**Table 4:** DB2 subject information sheet.

No	Laterality	Gender	Age	Height	Weight	No	Laterality	Gender	Age	Height	Weight
1	Right	Male	29	187	75	21	Right	Male	32	170	75
2	Right	Male	29	183	75	**22**	Left	Female	28	162	54
3	Right	Male	31	174	69	23	Right	Male	25	170	66
**4**	Left	Female	30	154	50	**24**	Right	Male	28	170	73
5	Right	Male	25	175	70	**25**	Left	Male	31	168	70
6	Right	Male	35	172	79	26	Left	Male	30	186	90
7	Right	Male	27	187	92	27	Right	Male	29	170	65
8	Right	Male	45	173	73	**28**	Right	Female	29	160	61
9	Right	Male	23	172	63	**29**	Right	Male	27	171	64
**10**	Right	Male	34	173	84	30	Right	Male	30	173	68
11	Right	Female	32	150	54	31	Right	Male	29	185	98
**12**	Right	Male	29	184	90	32	Right	Male	28	173	72
13	Left	Male	30	182	70	33	Right	Male	25	183	71
14	Right	Female	30	173	59	**34**	Right	Male	31	192	78
15	Right	Male	30	169	58	**35**	Right	Female	24	170	52
**16**	Right	Male	34	173	76	**36**	Right	Female	27	155	44
17	Right	Male	29	175	70	37	Right	Male	34	190	105
**18**	Right	Female	30	169	90	38	Right	Female	30	163	62
19	Right	Female	31	158	52	**39**	Right	Male	31	183	96
20	Right	Female	26	155	52	40	Right	Male	31	173	65

bold numbers indicate subjects with lower accuracy than baseline. Acc was marked as low.

**Fig 11:**
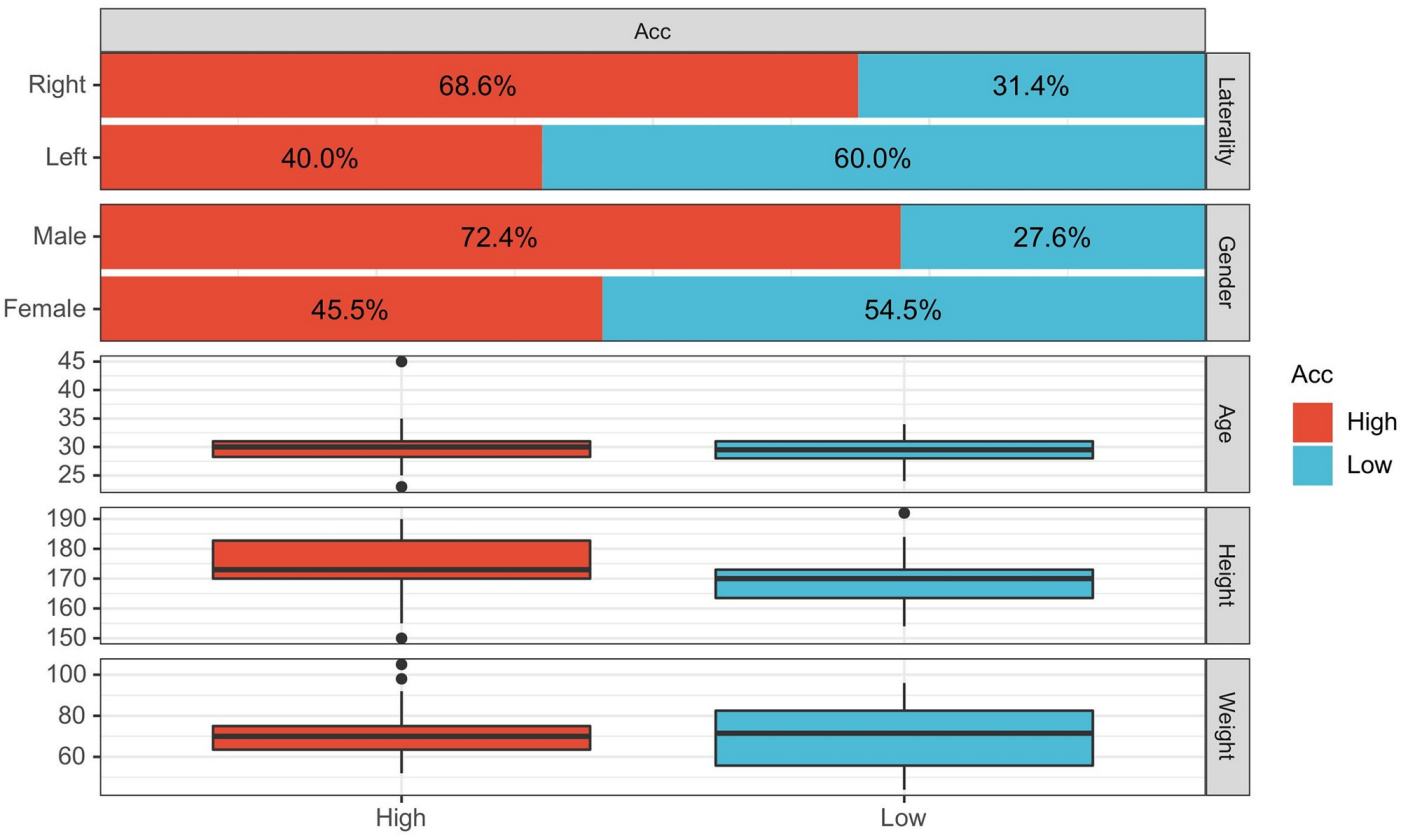
DB2 subject identification accuracy and information bivariate plot.

Finally, a bivariate graph is drawn according to the subject attribute information, as shown in Fig 11. It shows the number distribution of subjects whose ACC was marked as high and low on attributes such as Laterality, Gender, Age, Height, and Weight, and the accuracy shows significant differences in gender and preference hands.

Our inference is that the apparent difference in gender attribute accuracy may be because females are generally inferior to males in the intensity of action stimuli. Differences in the accuracy of the laterality attribute may be influenced by minority subjects and by motor differences between left-handed and right-handed individuals.

**Fig 12:**
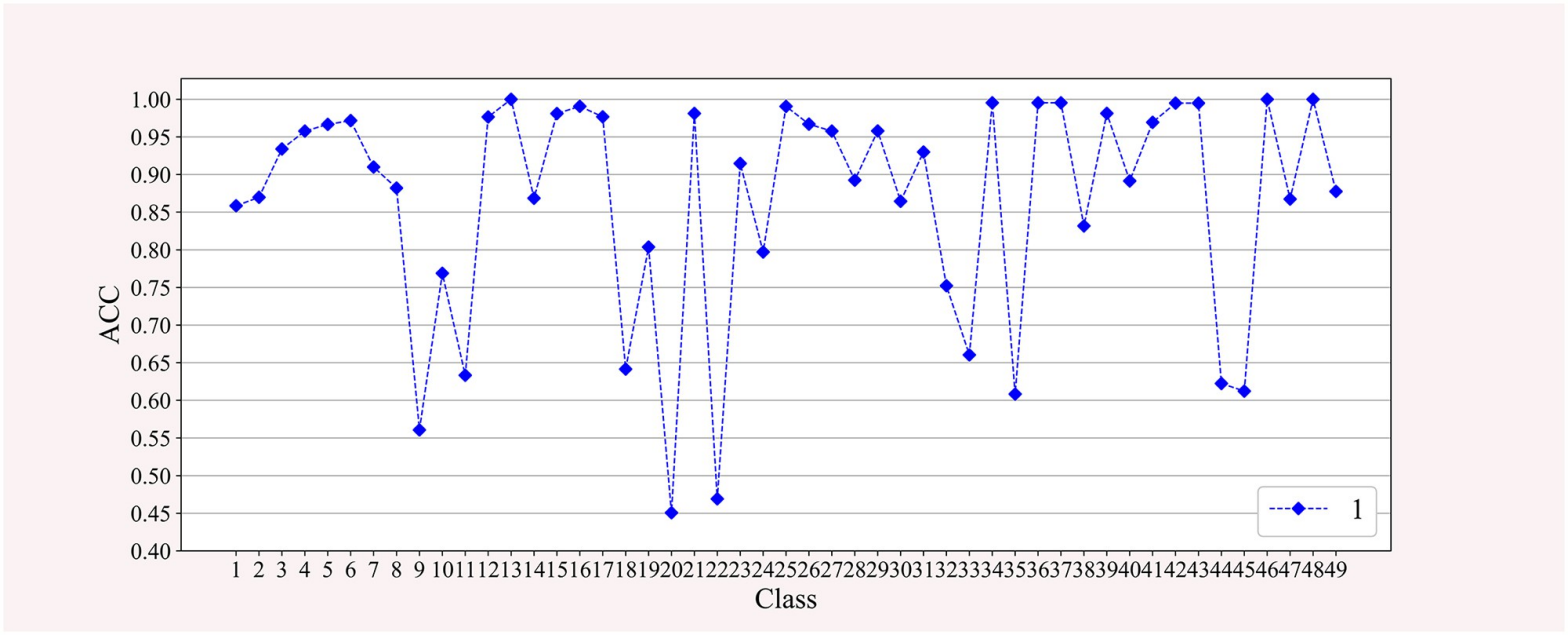
The recognition accuracy of 49 classes of movements of subjects.

To compare the difference in the accuracy of different action recognition, we randomly show the accuracy of 49 categories of action recognition in a cross-validation experiment for 1 subject in DB2. As shown in Fig 12, among actions with lower than average recognition accuracy, types 9, 10, and 11 are wrist rotations with high similarity. Types 18 and 22 are grasping plastic bottles of different sizes. Types 32, 33, and 35 are grasping small objects. In general, these movements have the characteristics of small stimulation and similar movements. It is difficult to further improve the recognition rate by analyzing only the sEMG collected by the arm. The Ninapro database also records electrode gloves and triaxial acceleration values. The research [35] combining the signals collected by different devices is an effective method to solve this problem.

**Fig 13:**
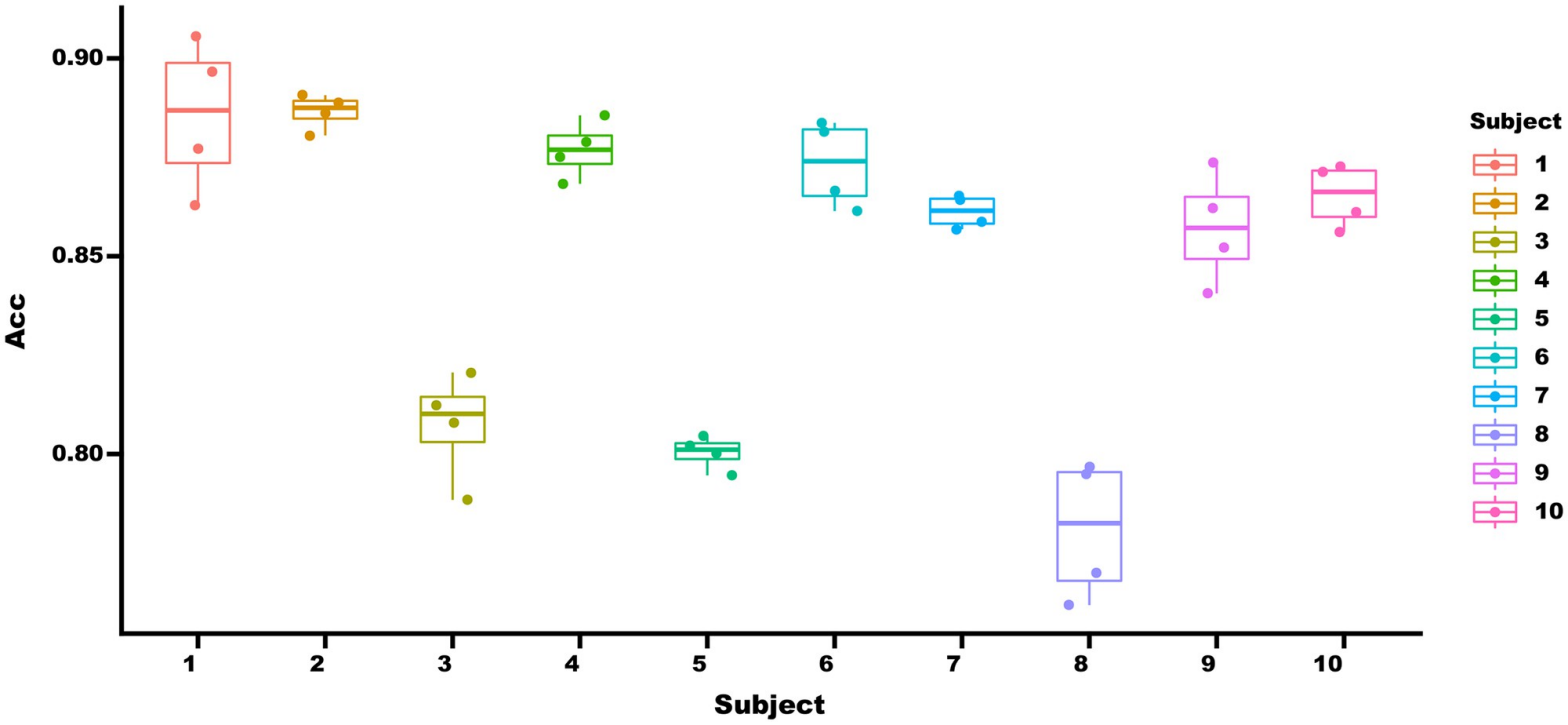
The average gesture recognition accuracy of DB4 subjects.

To test the effectiveness of our proposed method in more databases, we also conducted the same experiment on 10 subjects in the DB4 database, and the cross-validation results of repeated gestures four times on the training set are shown in Fig 13.

The cross-validation results verify the generalization ability of the proposed method on the DB4 database, and Table 3 presents the average results of four experiments. Comparing the acquisition methods of DB2 and DB4 databases, the difference in data level between the two is eliminated by Z-score standardization, because the electrode distribution and acquisition protocol are the same, the number of classified gestures is similar, and the final overall classification results are also similar, which meets the experimental expectations.

## 4. Discussion

In our proposed EMG gesture recognition network, the standardization of input data, the number and size of convolution kernels in the multi-stream convolution stage, the dropout rate in the early and late aggregation stages, and the weights of early fusion and late fusion all affect the final recognition accuracy. To determine the ideal parameter settings, we selected the raw label data of 3 subjects in the DB2 database as the base data and analyzed the effects using the same experimental settings. Where S1 is male, S26 is left-handed, and S38 is female. The subject’s classification Accuracy (Acc) was used as the evaluation metric.

### 4.1 BConv module settings effects

The general batch normalized convolution process order is a convolutional layer, BN layer, and activation function [36]. In our experiments, we found that the BN layer performs better after the ReLU activation function. The experimental results are shown in Table 5.

**Table 5:** The BN layer sequence experiment.

BConv module settings	Acc (%)		
	S1	S26	S38
Conv + BN + ReLU	75.31	76.79	81.41
Conv+ ReLU +BN	**87.57**	**87.31**	**89.41**

Analyzing the experimental results, we believe that some of the output of the features after convolution may be negative, and these features will be truncated by ReLU nonlinearity. If normalization is done before ReLU activation, including negative values in the normalization before culling from the feature space brings a suppressive effect. The activated BN will normalize the positive features and pass them to the lower convolution, without counting the unpassable features.

### 4.2 Early and late network weight effects

In our proposed EMG gesture recognition network, the global feature aggregation output stage performs feature-level fusion on the outputs of the two sub-networks of early fusion and late fusion. We obtained the results after multiple experiments as shown in Table 6.

**Table 6:** The influence of early and late network weights on classification performance.

Early and late network weight	Acc (%)		
	S1	S26	S38
*H*_*early*_ = 0, *H*_*late*_ = 128	87.36	86.47	88.95
*H*_*early*_ = 32, *H*_*late*_ = 128	87.57	87.31	89.41
*H*_*early*_ = 64, *H*_*late*_ = 128	85.29	87.62	89.26
*H*_*early*_ = 128, *H*_*late*_ = 128	88.31	86.47	88.72

### 4.3 Ablation studies

To better analyze the source of our results, we refer to the method of ablation experiments [37] to analyze the variables of the proposed model layer by layer. The results of the experiments (E1-E7) are shown in Table 7 below.

**Table 7:** The classification and performance comparison of Ablation studies.

Methods	Acc (%)		
	S1	S26	S38
E1: CNN	79.19	78.20	82.11
E2: BConv	85.89	85.28	87.83
E3: 12-stream BConv	86.80	84.55	88.69
E4: Multi-stream BConv	87.41	86.07	88.76
E5: E4+ResCBAM	87.36	86.47	88.95
E6: E5+ Early and late feature aggregation.	87.57	87.31	89.41
E7: E6+ Time-warped data enhancement	**89.52**	**87.76**	**89.34**

It can be seen from Table 7 that BConv has the greatest improvement in recognition accuracy, which is since batch normalization reduces the effect of internal covariate bias and normalizes the increasingly biased output distribution to the ideal range, allowing the activation input values to fall in sensitive regions, avoiding gradient disappearance while speeding up the training speed. In experiments 2, 3, and 4, we tested the scheme of all sensors as one input, the scheme of separate branches for each electrode sensor, and the scheme of dividing branches according to sensor distribution. Overall, our method is optimal, because the multi-stream convolution process adopts the divide-and-conquer idea for extracting the features of different muscles independently and prevents the interference of irrelevant muscles. Experiment 5 shows that the added ResCBAM sequentially derives the attention map along two independent dimensions of channel and space, and performs adaptive feature extraction, the new feature and the original feature increase the feature amount by adding, and the effect is improved. Experiment 6 shows that the added early and late feature aggregation network extracts early original features and late high-level semantic features, which further improves the recognition rate. Finally, time warping is added for data enhancement, which makes the data more diverse, prevents overfitting, and achieves the highest overall average recognition accuracy.

The multi-stream feature fusion network proposed in this paper uses multi-stream convolution and spatial attention, and according to the characteristics of multi-channel EMG signals, adaptively extracts the morphological features of the signal of a single electrode acquisition channel and the spatial features of multiple electrode acquisition channels at the same time. The feature channel attention mechanism is used to adaptively assign different weights to different feature maps, increase the weight of effective feature maps, suppress invalid feature maps, and obtain more detailed features. Finally, an aggregation network is used to aggregate early primitive features and late high-level semantic features to output classification results. The average recognition accuracy of 49 types of gestures on the stimulus label data reaches 87.02%, which brings optimization from the classification performance and data requirements.

### 4.4 Comparison of similar literature

We compare the recognition model in this paper with gesture recognition models that have been studied on the Ninapro database in recent years in Table 8, and our method excels in both the number of gesture classifications and the overall recognition accuracy (OA).

**Table 8:** Comparison of the classification performance by the proposed network and other methods.

Reference	Dataset	category	Methods	OA (%)
Hu [26]	DB2	50	CNN-RNN Attention	82.2
Zhang [23]	DB2	49	information bottleneck, SNN	75.4
Ding [38]	DB2	17	Multi-stream-CNN	78.86
Kim [39]	DB2	17	sEMG feature, CNN-LSTM	83.91
Tosin [40]	DB2	17	SVM-RFE, Monte Carlo, SVD entropy	84.3
Gulati [33]	DB2	17	Hybrid CNN-RNN	81.96
Wei [34]	DB2	50	3 views of sEMG, Multi-view CNN	85.8
	DB4	53	3 views of sEMG, Multi-view CNN	72.9
Wei [19]	DB2	50	3 views selected from 11 sEMG feature sets, Multi-view CNN	83.7
Our work	DB2	49	Multi-stream CNN+ ResCBAM	**87.02**
	DB4	52	Multi-stream CNN+ ResCBAM	**84.87**

Among them, Hu [26], Ding [38], Gulati [33], and others only focus on extracting the morphological or temporal features of the signal in the sliding window, ignoring the differences and connections between different electrode channels. The artificial features designed by Tosin [40], Kim [39], Wei [19], and others have high requirements on researchers’ experience, and to some extent destroy the hidden connections between real signals.

Compared to these studies using the Ninapro database, our network does not need a professional artificial feature set design, directly uses the original EMG signal as input, and uses four-time cross-validation of repeated gestures on the training set to make the results more reliable. But nearly a quarter of the real data in the cross-validation process is not directly involved in network training, so we enrich the training set with time-warped data augmentation. We excluded rest states in the number of classified gestures because most of the existing studies in the Ninapro database [26, 32, 35–37] excluded rest, which is the easiest to distinguish and accounts for half of the total number of movements, accounting for half of the total number of movements, but not the focus of the study. In addition, restimulus recordings marked some missing EMG data as rest, and these erroneously labeled data could not be used to examine network performance. Finally, good classification results are obtained on differently labeled data and different databases. Compared with similar methods, this method has better recognition performance.

In these studies using other databases, Compared to [20], the input of MSFF-Net is not the whole gesture, it can recognize actions with large differences in duration. Compared to [21], the BConv stage uses a 2D narrow convolution kernel to extract the time and feature information of different electrode sensor channels, respectively. Compared to [22], MSFF-Net discusses the advantages of sensor shunting by region over input shunting and individual shunting with ablation experiments.

## 5. Conclusion

In this paper, a novel MSFF-Net sEMG signal gesture recognition model is proposed. The biggest feature of this model is that the signals collected in different areas are analyzed separately according to the electrode position, which can more fully extract the features of the multi-channel sEMG signal. In the signal preprocessing stage of this method, the denoised sEMG signal is segmented to ensure the independence of each action, and then the order of magnitude of the signal is changed by Z-score standardization to facilitate feature extraction. Finally, sliding segmentation is performed on the action segment to identify the signal from the perspective of the image. In the feature extraction stage, the multi-stream convolution network isolates the signal interference of different muscle regions and retains the signal correlation of the same muscle region. ResCBAM module further extracts deep features from signal shape, electrode acquisition space, and feature space, and the early-late aggregation network integrates the original features after signal standardization and the high-level semantic features of multi-stream convolution. Experiments show that the average recognition accuracy of the proposed model for 49 types of gestures of 40 healthy subjects in NinaPro’s DB2 database and 52 types of gestures of 10 healthy subjects in the DB4 database is better than the existing similar methods. The proposed network model helps to improve the accuracy of gesture recognition based on sEMG and provides a new idea for the current research of human-computer interaction based on sEMG. In the process of motion acquisition of sEMG signal, the phenomenon of sensor dislocation or movement is inevitable, which may affect the performance of the reference electrode arrangement classification method proposed in this paper. In the follow-up research, we will further try to solve this problem by combining feature set images and signal images.
